# Research progress on extraction, separation, structure, and biological activities of polysaccharides from jujube fruit (*Ziziphus jujuba* Mill.): a review

**DOI:** 10.3389/fchem.2025.1581947

**Published:** 2025-04-16

**Authors:** Feilong Jia, Bo Wang, Hui Ma, Changcai Bai, Yuanyuan Zhang

**Affiliations:** ^1^ Department of Pharmacy, General Hospital of Ningxia Medical University, Yinchuan, China; ^2^ Department of Pharmacy, People's Hospital of Ningxia Hui Autonomous Region, Ningxia Medical University, Yinchuan, China; ^3^ College of Pharmacy, Ningxia Medical University, Yinchuan, China; ^4^ School of Chinese Materia Medica, Beijing University of Chinese Medicine, Beijing, China

**Keywords:** *Ziziphus* jujuba polysaccharides, pharmacological activities, structure-activity relationship, structural characteristics, *Ziziphus jujuba* Mill

## Abstract

Jujube (*Ziziphus Jujuba* Mill.) is an excellent medicinal and edible plant owing to its high nutritional and health-promoting properties. As an important bioactive component, *Z*. Jujuba polysaccharides have aroused wide attention due to their various pharmacological activities, including anti-inflammatory, immunomodulatory, anti-oxidant, anti-tumor, anti-viral, regulating gut microbiota, hepatoprotective effects and prebiotic activity, and so on. This review highlights the advancements in the extraction methods, structural characteristics, structural elucidation, and functional analysis of polysaccharides derived from Jujube fruits over the past decade, aiming to provide valuable insights for future development and commercialization of Jujube fruits polysaccharides.

## 1 Introduction

**FIGURE 1:**
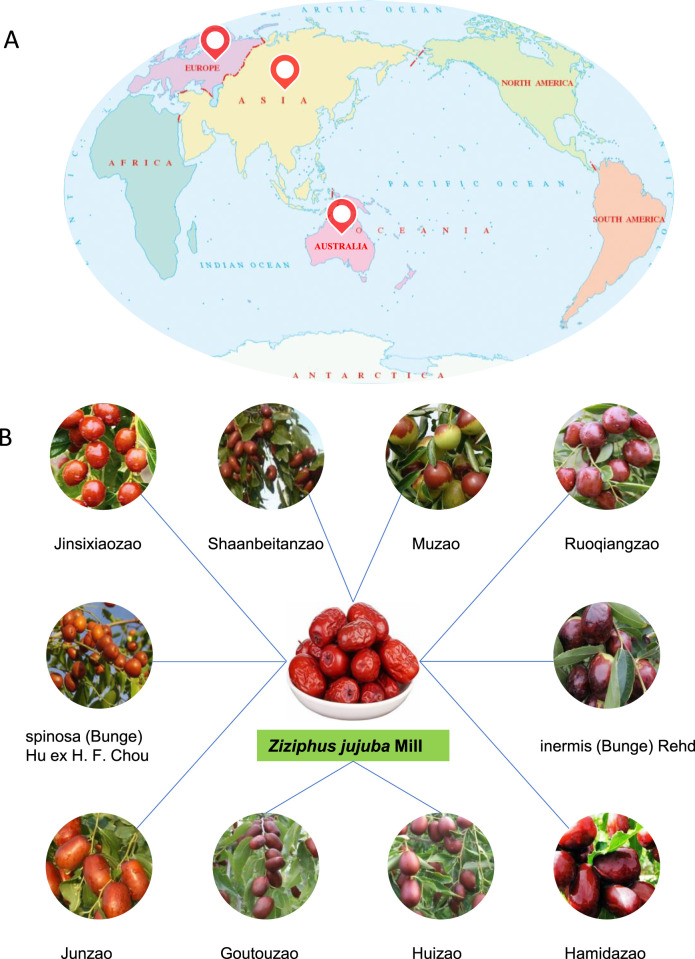
The distribution of *Ziziphus jujuba* Mill **(A)** (https://www.gbif.org/species/2536891), and the source of *Ziziphus jujuba* Mill **(B)**.

Jujube (*Ziziphus Jujuba* Mill.) is a small deciduous tree that belongs to the genus Ziziphus (Rhamnaceae family), is native to China with over 7,000 years of cultivation history, and is extensively distributed in Asia, Europe, and Australia (Figure 1A), mostly in Xinjiang, Hebei, Shandong, Shaanxi, Henan, Ningxia, Shanxi, and other places in China (Gao et al., 2013; LIU et al., 2015b; Liu et al., 2024). There are different jujube trees around the world, most of which have been extensively cultivating in China, including *Ziziphus jujuba* cv Jinsixiaozao, *Ziziphus Jujuba* cv. Muzao, *Ziziphus Jujuba* cv. Huizao, *Ziziphus Jujuba* cv. Shaanbeitanzao, *Ziziphus Jujuba* var. spinosa (Bunge) Hu ex H. F. Chou, *Ziziphus Jujuba* cv. Ruoqiangzao, *Ziziphus Jujuba*. var. inermis (Bunge) Rehd, *Ziziphus Jujuba* cv. Junzao, *Ziziphus Jujuba* cv. Hamidazao, *Ziziphus Jujuba* cv. Huanghetanzao, etc., (Figure 1B). It is worth mentioning that the edible part of the plant is generally the fruits of *Ziziphus Jujuba* Mill. has been extensively used as a medicinal and functional fruit. The medicinal history of *Ziziphus Jujuba* Mill. first appeared in Huangdi Neijing (475–221 BCE) to treat various diseases two thousand years ago. The historical documentation of jujube application can be traced back to these monumental works such as Shanghanzabinglun, Mingyibielu, Compendium of Materia Medica, Chinese Medicine Dictionary, Chinese Herbal Medicine, and Chinese Pharmacopoeia (Chen et al., 2015; Chen et al., 2014). In traditional Chinese medicines, *Z*. Jujuba has the favorable functions of strengthening sleep quality, improving food digestion, delaying the life-span, and nourishing the body. In recent years, a large number of attentions have paid to the chemical components, biological activities and application of *Z*. Jujuba. Notably, as a homology of medicine and food, *Ziziphus Jujuba* Mill. comprises abundant nutrients: polysaccharides, proteins, fatty acids, flavonoids, dietary fiber, triterpenoids, and vitamins, and other active constituents (Liu M. et al., 2020; Wojdyło et al., 2016), which demonstrates heterogeneous pharmacological activities, containing neuroprotective, anti-tumor, anti-inflammatory and antioxidant, immune-stimulation, liver protection (Bahrami et al., 2024; Șumălan et al., 2025; Xiao et al., 2022; Zou et al., 2024). Polysaccharides, among these components, are deemed as one of the main bioactive compounds of *Z*. Jujuba (total content 21.9 g/100 g), their biological activities have attracted increasing attention in decades (Ji et al., 2023). For decades, numerous investigations have been conducted to explore the pharmacological properties and prospective applications of *Z*. Jujuba polysaccharides. It is noteworthy that Jujuba polysaccharide exhibited satisfactory physicochemical properties and an abundance of biological activities, involving anti-inflammatory, immunomodulatory, anti-oxidant, anti-cancer, anti-viral, gut microbiota regulation, hepatoprotective effects and prebiotic activity, demonstrating multifarious benefits to human health (Han et al., 2024; Hua et al., 2022; Ji et al., 2019a). Polysaccharides from distinct natural products have different biological activities due to their unique structures, and this activity varies with glycoside bond, repeat unit, monosaccharide content, space conformation, linking group, and molecular weight. Different extraction and purification methods of *Z*. Jujuba have an impact on the structures and biological activities of its polysaccharides. As a result, the *Z*. Jujuba polysaccharides extracted by various procedures must be recognized for their specific structures, and their corresponding structures need to be thoroughly investigated in terms of biological efficacy and molecular mechanisms. At the moment, investigation into *Z*. Jujuba polysaccharides remains in its early stages. Although various biological effects and beginning mechanisms have been discovered, numerous issues remain, including the impact that extraction on the structure of polysaccharides and the structure-activity correlation of polysaccharide. This paper reviews the separation, chemical structures, and pharmacological properties of Jujuba polysaccharides, with the objective of offering references for subsequent investigation and application.

## 2 Extraction, isolation and purification of jujuba polysaccharides

**FIGURE 2:**
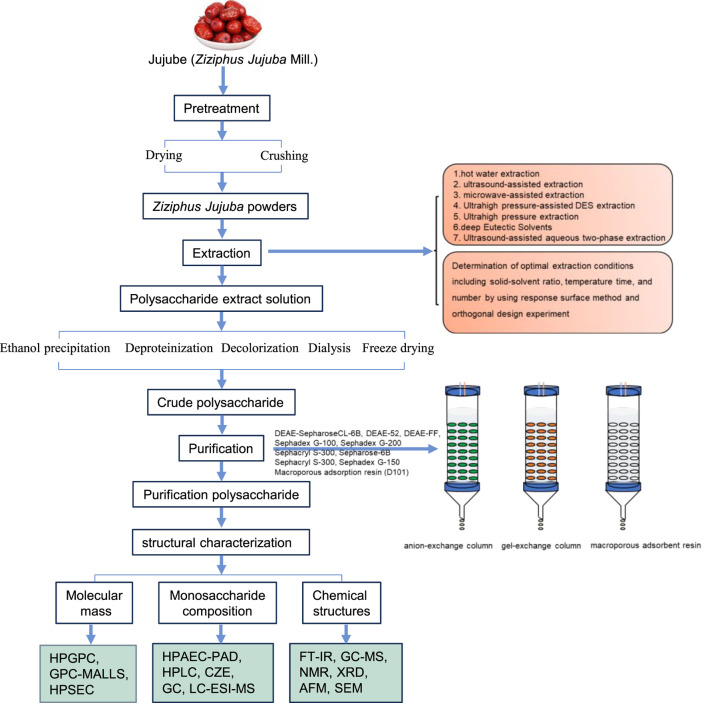
Extraction, purification, and structural characterization of polysaccharides from *Z. jujuba* Mill.

Polysaccharides are both polar and hydrophilic macromolecules made up of the same or various monosaccharide units connected by glycosidic linkages. They are often soluble in water but insoluble in organic solvents. Polysaccharides are considered a substantial component of the *Z*. jujuba fruit, and their extraction and purification have received increasing attention. A schematic diagram is shown in Figure 2.

### 2.1 Extraction methods

Extraction is an important stage in polysaccharide investigation since it allows for the isolation of polysaccharides for further structural and pharmacological analysis, establishing the framework for the creation of new medicinal agents and health supplements. Developments in science and technology have resulted in considerable advances to extraction and purification processes. Given the increased knowledge of the nutritional and therapeutic benefits of traditional Chinese medicine polysaccharides (TCMPs), there has been an abundance of interest in researching these molecules. Over the last few decades, multiple techniques have been used to extract polysaccharides from *Z. jujuba* fruit, highlighting the significant potential of jujube polysaccharides as valuable natural compounds and advocating for their further exploration and application in industrial and therapeutic settings. Table 1 summarizes the different extraction, purification methods, and the corresponding *Z. jujuba* fruit yield. Prior to the extraction of polysaccharides, *Z. jujuba* fruit undergo a series of pretreatment steps are generally required, mainly including milling the fruits into a fine powder (Liu et al., 2020b), followed by removing the lipids and pigments either by soaking in 85% ethanol or H_2_O_2_ at 50°C for 1.5 h (Guo et al., 2019; Zhao et al., 2008). Moreover, the fruits could also be defatted with petroleum ether or other organic solvents (Lin et al., 2018). These pre-treatment methods effectively remove pigments of the fruits and the solid residue is used for subsequent reflux extraction.

**TABLE 1:** Extraction, pre-treatment, and yield of PNPs.

Name	Polysaccharides source	Extraction	Pretreatment	Time (min)	Temp (°C)	Power	Solid liquid ratio	Optimization method	Yield (%)	Purification methods	Ref
ZSP	Z. jujube cv. Shaanbeitanzao	HWE	The fruit was dried in a microwave oven, then crushed and extracted with water	160	80	—	1:20, w/v		7.2	Ethanol precipitation, freeze–thaw process protein removal, dialysis	Wang et al. (2012)
CZSP	Zizyphus jujuba cv. Jinsixiaozao	HWE	fruits were pre- extracted with 95% ethanol	180	80	—	—		—	DEAE-SepharoseCL-6B anion-exchange column	Li et al. (2011)
ZSS	Ziziphus jujuba Mill var. spinosa (Bunge) Hu ex H. F. Chou	HWE	Powders were degreased of petroleum ether	180	70		7.5 mL/g		0.93	Macroporous adsorption resin (D101) and anion exchange resin remove the pigment and protein	Lin et al. (2018)
JP	Zizyphus jujube	HWE	Powders were prepared	180	70		1:15			Pigments were removed by hydrogen peroxide, Sevage protein removal, dialysis	Guo et al. (2019)
WSPs	Ziziphus jujuba Mill.	HWE	fruits were pre- extracted with 85% ethanol	240	100				5.1	Ethanol precipitation, chloroform–butyl alcohol protein removal	Zhao et al. (2008)
—	Ziziphus jujuba Mill	HWE	Powders were prepared, powder were pre- extracted with 80% ethanol	270	100	—	—	—	—	Ethanol precipitation, Sevage protein removal	Ji et al. (2017)
ZP2a	Zizyphus jujuba cv. Junzao	HWE	Powders were prepared, powder were pre- extracted with 80% ethanol	180	80	—	—	—	—	Ethanol precipitation, dialysis, DEAE-SepharoseCL-6B	Li et al. (2013)
ZMP	Ziziphus Jujuba cv. Muzao	HWE	fruits were pre- extracted with 80% ethanol	—	—	—	—	—	—	Ethanol precipitation, Sevage protein removal, hydrogen peroxide solution decolorization, dialysis, DEAE cellulose-52 and Sephadex G-100 size-exclusion chromatography	Ji et al. (2018a)
ZJP	Ziziphus jujuba Mill (Zizyphus jujuba cv. Junzao)	HWE	Powders were prepared	240	90	—	—		0.17	Ethanol precipitation, Sevage protein removal, activated carbon decolorization, DEAE-52 cellulose chromatography column, Sephadex G-200 gel permeation column chromatography	Zhan et al. (2018)
JU	Ziziphus jujuba Mill. cv. jinsixiaozao	HWE	Fruits were pre- extracted with ethanol	240	100					Ethanol precipitation, chloroform-butyl alcohol protein removal, DEAE chromatography, Sepharose CL-6B chromatography	Zhao et al. (2006)
JP-H	Ziziphus jujuba Mill.	HWE	Fruits were pre- extracted with ethanol, Powders were prepared	120	90		1:30		3.12	—	Zou et al. (2022)
PZMP2-2	Ziziphus Jujuba Mill	HWE	Powders were prepared	—	—	—	—	—	14.15	DEAE-Sepharose FF column, Sephacryl S-300 column chromatography	Ji et al. (2019c)
LZJPs	Ziziphus jujuba Mill.	HWE	Powders were prepared	90	80		1:20	Single-factor test, Box–Behnken Design matrix	5.04	Sevage protein removal, DEAE-52 cellulose column, Sephadex G-100 column chromatography	Wang et al. (2018)
CZP	Zizyphus jujuba cv. Junzao	HWE	Powders were prepared, Powders were pre- extracted with 80% ethanol	180	80	—	—	—		Ethanol precipitation, dialysis, DEAE-SepharoseCL-6B column, gel-filtration column	Li et al. (2013)
-	Ziziphus jujuba Mill. var. inermis (Bunge) Rehd	HWE	Powders were prepared, Powders were pre- extracted with 95% ethanol	300	100	—	—	—	—	Ethanol precipitation	Wang (2011)
ZSP	Zizyphus jujube cv. Shaanbeitanzao	HWE	Powders were prepared	160	80	—	1:20		7.2	Ethanol precipitation, dialysis	Zhao et al. (2014)
ZMP	Ziziphus Jujuba cv. Muzao	HWE	Powders were prepared	30	70	415	1:20			Ethanol precipitation, DEAE-Sepharose Fast Flow and Sephacryl S-300 columns	Ji et al. (2020a)
CZPH	Zizyphus Jujuba cv. Junzao	HWE	Fruits were pre- extracted with ethanol	180	80				6.23	—	Li et al. (2014)
JP	Zizyphus jujuba Mill. Goutouzao	HWE	The fruits were Minced, fruits were pre- soaked with hot water	60	90	—	—	—	3	Ethanol precipitation, trichloroacetic acid protein removal, Hydrogen peroxide decolorization, dialysis	Xu et al. (2022)
HP	Zizyphus jujuba cv Huizao	HWE	Powders were prepared	360	70		1:50			Ethanol precipitation, Sevage protein removal, dialysis, DEAE-52 cellulose column, Sephadex G-200 gel column	Zou et al. (2018)
PWJS	Ziziphus jujuba Mill. var. spinosa (Bunge) Hu ex H. F. Chou	HWE	Powders were prepared, Powders were pre- extracted with 95% ethanol	540	80	—	1:25	—	9.8	Ethanol precipitation, dialysis	Yue et al. (2014)
JPC	Ziziphus Jujube	HWE	Powders were prepared, Powders were pre- extracted with 95% ethanol	180	80		1:10	—	3.16	Ethanol precipitation, Sevage protein removal, dialysis, DEAE-52 cellulose column, Sephadex G-150 gel column	Chi et al. (2015)
ZMP	Ziziphus Jujuba cv. Muzao	HWE	—	—	—	—	—	—	3.82	DEAE-Sepharose Fast Flow, Sepharcyl S-300 gel-filtration column	Ji et al. (2022)
BJPs	Z. jujuba cv. Hamidazao	HWE	Powders and slice were prepared	45	80		1:10		2.3	Ethanol precipitation, dialysis, ethyl acetate and Sevage protein removal, DEAE-cellulose 52 anion-exchange column, Sephadex G-100 gel column	Yuan et al. (2022)
WSPJ	Ziziphus jujuba Mill	HWE	Powders were prepared	180	60		1:20	Single-factor test, Box–Behnken Design matrix			Qu et al. (2013)
HJP	Z. jujuba cv. Hamidazao	HWE	Powders were prepared	90	—	—	1:10	—	2.3	Ethanol precipitation, dialysis, Sevage protein removal, Petroleum ether degreasing, DEAE-cellulose 52 anion-exchange column, Sephadex G-100 gel column	Yang et al. (2021)
ZMP	Ziziphus jujuba cv. Muzao	HWE	Powders were prepared						3.82	Ethanol precipitation, DEAE-Sepharose, Sephacryl S-300	Ji et al. (2023)
ZSS	Ziziphus jujuba Mill var. spinosa (Bunge) Hu ex H. F. Chou	UAE	Powders were degreased of petroleum ether	21.2	52.5	134.9 W	26.3 mL/g	Single-factor test, Box Behnken design response surface	1.05	Macroporous adsorption resin (D101) and anion exchange resin remove the pigment and protein	Lin et al. (2018)
ZJPs-II	Ziziphus jujuba (Z. jujube cv. Ruoqiangzao)	UAE	Powders were prepared	100	83	140 W				ethanol and acetone precipitation, Sevage protein removal, dialysis, DEAE-52 column, Sephadex G-100 column	Wu et al. (2021)
ZMP	Zizyphus jujube cv. Muzao	UAE	fruits were pre- extracted with 80% ethanol	30	70	415 W	23:1			Ethanol precipitation, Sevage protein removal	Ji et al. (2017)
PZMP4	Ziziphus jujuba Mill (Ziziphus Jujuba cv. Muzao)	UAE	—	—	—	—	—	—	—	Ethanol pre-cipitation, deproteination, dialysis, DEAE-Sepharose Fast Flow, Sephacryl S-300 column chromatography	Ji et al. (2021a)
HJPs	Ziziphus jujuba Mill (Ziziphus Jujuba cv. Muzao)	UAE	—	—	—	—	—	—	—	Sevage protein removal, hydrogen peroxide solution decolorization, DEAE-Sepharose Fast Flow column	Wang et al. (2015)
HJP	Ziziphus jujuba Mill cv. Muzao	UAE	Powders were prepared, Powders were pre- extracted with 95% ethanol	15	50	—	—	—	9.8	Ethanol precipitation, Sevage protein removal, DEAE-Sepharose fast flow column	Zhang et al. (2017)
JP-UD	Ziziphus jujuba Mill.	UPADESE	fruits were pre- extracted with ethanol	8.25		483	1:3.75		10.42	—	Zou et al. (2022)
HJP	Zizyphus jujube cv. Huanghetanzao	UAE	Powders were prepared, Powders were pre- extracted with 95% ethanol	15	50	—	—	—	—	Ethanol precipitation, dialysis	Liu et al. (2015a)
CZPU	Zizyphus Jujuba cv. Junzao	UAE	fruits were pre-extracted with ethanol	20	50				7.95	—	Li et al. (2014)
WSPJ	Ziziphus jujuba Mill	UAE	Powders were prepared	15	40	100	1:20	Single-factor test, Box–Behnken Design matrix			Qu et al. (2013)
JCP	Z. jujube Mill	MAE	—	60	75	400	1:30	Single-factor test, Box–Behnken Design matrix	9.02	Ethanol precipitation, dialysis, DEAE-cellulose 52 column, Sephadex G-200 column	Rostami and Gharibzahedi (2016)
WSPJ	Ziziphus jujuba Mill	MAE	Powders were prepared	4	—	400	1:20	Single-factor test, Box–Behnken Design matrix			Qu et al. (2013)
AJP	Jinsixiaozao	alkali-extracted	The fruits were dried, crushed, and passed through a 60-mesh Sieve	120	80	—	1:30	—	—	Ethanol precipitation, membrane separation	Wang et al. (2023)
ZY	Ziziphus jujuba Mill. var. Spinosa (Bunge) Hu ex H. F. Chou	alkali-extracted	Powders were prepared	141.6	—	—	—	1:16.56	1.98	Ethanol precipitation	Liu et al. (2023)
SAZMP	Ziziphus jujuba cv. Muzao	alkali-extracted	Powders were prepared, Powders were pre- extracted with 50°C water	60	30		1:10		3.3	Ethanol precipitation, Sevage protein removal, Hydrogen peroxide decolorization, DEAE-Sepharose Fast Flow column, Sephacryl S-300 gel-filtration column	Lin et al. (2019)
SAZMP4	Ziziphus jujuba cv. Muzao	alkali-extracted	—	—	—	—	—	—	5.3	DEAE-Sepharose Fast Flow, Sepharcyl S-300 gel-filtration column	Lin et al. (2020)
ZJRP	Ziziphus jujuba cv. Muzao	alkali-extracted	Powders were prepared		90		1:20	Single-factor test, Box–Behnken Design matrix	5.1	Hydrogen peroxide decolorization	He et al. (2019)
AZMP	Ziziphus jujuba cv. Muzao	alkali-extracted	Powders were prepared						3.30	Ethanol precipitation, DEAE-Sepharose, Sephacryl S-300	Ji et al. (2023)
SAZMP3	Zizyphus jujuba cv. Muzao	alkali-extracted	Powders were prepared	60	30		1:10	—	3.3	Ethanol precipitation, Sevage protein removal, hydrogen peroxide decoloration, DEAE-Sepharose Fast Flow column, Sephacryl S-300 gel-filtration column	Lin et al. (2019)
ZJSPs-1	Z. jujube cv. Ruoqiangzao	UAHE	Powders were prepared	100	83.1	140	1:33.5	response surface methodology	1.97	DEAE 52-cellulose column, Sephadex G-100 column	Wu et al. (2019)
JP-U	Ziziphus jujuba Mill.	UPE	Fruits were pre- extracted with ethanol, Powders were prepared	8.25		483	1:30		7.98	—	Zou et al. (2022)
JP-D	Ziziphus jujuba Mill.	DES	Fruits were pre- extracted with ethanol, Powders were prepared	120	90		1:30		6.36	—	Zou et al. (2022)
ZMP	Ziziphus jujuba Mill. cv. Muzao	UAATPE	Powders were prepared, Powders were pre- extracted with ethanol	38	40	70	1:30	Single-factor test, Box–Behnken Design matrix	8.18	—	Ji et al. (2018b)
CZP	Ziziphus jujuba Mill.	subcritical water	Powders were prepared	60	140			Single-factor test	7.9	Ethanol precipitation, Sevage protein removal, DEAE-cellulose 52 column	Liu et al. (2020c)

NOTE: Hot water extraction (HWE), Ultrasound-assisted extraction (UAE), Microwave-assisted extraction (MAE), Ultrahigh pressure-assisted DES, extraction (UPADESE), Ultrahigh pressure extraction (UPE), Deep eutectic solvent (DES), Ultrasound-assisted aqueous two-phase extraction (UAATPE).

Traditionally, the primary extraction technique for *Z. jujuba* polysaccharides involves solvent extraction, utilizing solvents such as hot water, alkaline solutions, which yield varying amounts of *Z. jujuba* polysaccharides depending on the solvent used. However, various eco-friendly and green technologies, such as microwave-assisted extraction (MAE), ultrasonic-assisted extraction (UAE), ultrasonic-enzyme assisted extraction (UEAE), ultrahigh pressure-assisted DES extraction (UPADESE), ultrahigh pressure extraction (UPE), deep eutectic solvent (DES), ultrasound-assisted aqueous two-phase extraction (UAATPE) have been increasingly reported for the efficient and selective extraction of *Z. jujuba* polysaccharides. Currently, hot water extraction (HWE) is a widely employed and accessible extraction method, offers advantages such as low cost and simplicity, which is generally employed for obtaining polysaccharides from natural resources in laboratory and industrial applications. Nevertheless, it has disadvantages like low extraction purity, high temperatures, and prolonged processing times. To increase extraction amount, fruits are usually crushed into powder beforehand, necessitating proper particle size selection; extremely fine powder forms colloids in extraction, delaying transfer, and filtration, perhaps resulting in polysaccharide loss. Zhan et al. demonstrated that polysaccharide ZJP extracted from *Zizyphus jujuba* cv. *Junzao* using water extraction, ethanol precipitation, savage protein removal, and activated carbon decolorization yielded 0.17% (Zhan et al., 2018).

Furthermore, dilute alkali techniques, another conventional extraction method, can improve the extraction efficiency and decrease the separation time of jujuba polysaccharides to a particular degree. Nevertheless, alkaline conditions must be rigorously managed to prevent the structural damage of jujube polysaccharides. Yue et al. demonstrated that polysaccharide WJPs extracted from *Z. jujuba* cv. *Muzao* using alkali extraction, ethanol precipitation, savage protein removal, and hydrogen peroxide decolorization yielded 3.3% (Lin et al., 2019).

In addition, another study demonstrated that UAE and HWE were employed to extract polysaccharide from *Zizyphus jujuba* cv. *Junzao*. They compared the yield, physicochemical properties, structural properties, and biological activities of these extracts. It was found that UAE altered several characteristics of CZP compared to HWE. Specifically, the yield of polysaccharides via UAE increased from 6.23% to 7.95%, which represents an increase of about 27.6%. Conversely, the carbohydrate content decreased by 82.3% and 78.7%. (Li et al., 2014).

MAE has been employed as a supplementary extraction method to improve the solid-liquid extraction process since the past few years. Research indicates that MAE is effective in extracting polysaccharides from *Z. jujube* Mill fruits. An orthogonal design confirmed that a material-to-liquid ratio of 1:30, microwave power of 400 W, and an extraction time of 60 min yielded a 9.02% extraction rate (Rostami and Gharibzahedi, 2016).

Subcritical water extraction has recently been recognized as a beneficial polysaccharide extraction technology due to its high yield, inexpensive price, simplicity of usage, and friendly to the environment. Liu et al. selected the optimal extraction process for supercritical water extraction through single factor experiments, which was: extraction time of 60 min, 140°C, liquid-solid ratio of 20 mL/g, polysaccharide extraction rate of 7.9%, significantly higher than the traditional HWE extraction of 6.76% and MAE extraction of 7.2% (Liu et al., 2020c). Thus, subcritical water extraction is a rapid and efficient methods.

The ultrahigh pressure extraction (UPE) is a complementary technological advances that has appeared in recent decades, which understands rapid, cost-effective, highly precise, and little solvent consumption, and solves the shortcomings of traditional methods of extraction like soaking, maceration, water percolation, and Soxhlet extraction, etc., (Xi, 2017). Zou et al. reported that the comparison among UPE, HWE and deep eutectic solvent extract (DESE) of Polysaccharide from *Zizyphus jujube* cv. *Hui-zao* was carried out. Among these four extraction methods, the extraction yield of UPE (7.98% ± 0.13) was significantly higher than HWE (3.12% ± 0.24) and DESE (6.36% ± 0.43). While the extraction time of UPE (8.25 min) was much shorter than the extraction time of DESE (120 min) and HWE (120 min) as well. In addition to using one method alone, there are also ways to combine the two methods. Zou et al. combined UPE with DESE, achieving an extraction rate of 10.42% ± 0.28% for JPs. Through characterization using scanning electron microscopy (SEM), High performance liquid chromatography (HPLC), Fourier transform infrared spectroscopy (FT-IR), and Nuclear magnetic resonance (NMR), they confirmed that the UPE/DESE method does not degrade polysaccharides, offering a high extraction rate and short extraction time. This method shows potential to replace traditional hot water extraction as a simple and efficient industrial extraction method (Zou et al., 2022).

With developments in extraction technology, various combinations of extraction methods, including ultrasound-assisted aqueous two-phase extraction (UAATPE), have emerged as viable alternatives to conventional polysaccharide extraction methods from *Ziziphus jujuba* Mill. Ji et al. (2018b) extracted *Z. jujube* polysaccharides by using UAATPE and optimized the extraction process via RSM based on single factor experimental results. The results show that the optimal combination of process parameters was as follows: extraction temperature of 48°C, and extraction time of 38 min, microwave power of 70 W, a solid-to-liquid ratio of 1 g–30 mL, and the yield of polysaccharides was 8.18% under the optimal extraction conditions.

### 2.2 Isolation and purification methods

After extraction, jujuba polysaccharides usually remain with many impurities, including inorganic salts, oligosaccharides, proteins, and lignin. The presence of these impurities will directly impact on the following purification, structural characterization, physicochemical properties, and biological function of jujuba polysaccharides, rendering it challenging to assess the link between the structure and function of polysaccharides. As a result, prior to separating and purifying polysaccharides, certain steps are required to eliminate non polysaccharide impurities, such as proteins and pigments, from the crude polysaccharides.

The elimination of proteins in crude jujube polysaccharides is frequently evaluated by Sevag, a repeated freeze-thaw method, trichloroacetic acid, and chloroform–butyl alcohol methods. Pigments can oxidize jujuba polysaccharides, thus affecting the chromato-graphic analysis and compromising an accurate identification of the polymer; in view of this, the removal of pigments is an essential step during the separation process. Activated carbon, microporous adsorption resin, and hydrogen peroxide solution are used to remove pigments.

To obtain homogeneous polysaccharides, crude jujuba polysaccharides should be further separated and purified. Column chromatography is an effective method for purifying natural components. In general, two types of column chromatography methods, ion exchange chromatography and the gel filtration chromatography are frequently employed to purify crude polysaccharides. Ion-exchange chromatography can separate neutral polysaccharides and acidic polysaccharides via gradient salt elution, whereas gel filtration can separate polysaccharides of different molecular weights (Mw). Ji et al. (2020a) using DEAE-Sepharose Fast Flow column (2.6 × 100 cm), neutral polysaccharides together with acidic polysaccharides were obtained by eluting with distilled water and different concentrations of NaCl gradient. Three main fraction ZMP1, ZMP2, ZMP3 was further separated by a Sephacryl S-300 column (2.6 cm × 100 cm) with deionized water at a flow rate of 0.8 mL/min, to obtain three homogeneous polysaccharide PZMP1, PZMP2 and PZMP3. At present, the commonly used anion exchange chromatographic columns for jujube polysaccharides are the DEAE-cellulose 52 column (Liu et al., 2020c; Wu et al., 2021), DEAE-Sepharose CL-6B (Li et al., 2013; Li et al., 2011) and DEAE-Sepharose fast flow column (Ji et al., 2019b; Zhang et al., 2017). The commonly used gel chromatographic columns are mainly the Sephacry (Ji et al., 2020a; Ji et al., 2019c), Sepharose (Ji et al., 2021a) and Sephadex (Rostami and Gharibzahedi, 2016; Wang et al., 2018). Dialysis, concentration and lyophilize treatment of jujube polysaccharides can be used for purification after column chromatography for better storage. The differences in purification processes result in significant variations in the monosaccharide composition, Mw, and main chain structure of jujube polysaccharides (Ji et al., 2020a; Wang et al., 2023; Zou et al., 2018). Therefore, the separation and purification of polysaccharides is very complex, and researchers must carefully choose appropriate separation and purification methods based on the characteristics of the polysaccharides being studied.

## 3 Chemical composition and structures of jujube polysaccharides

According to studies, the biological activity of jujube polysaccharides is significantly affected by their complex physicochemical properties, such as Mw, chain length, type and quantity of functional groups, type of glycosidic bonds, monosaccharide composition, molar ratio, and branching degree, which can be assessed using a combination of chemical and physical analysis methods. To date, literature data provide diverse results, nearly 71 polysaccharides have been obtained and purified from *Ziziphus jujuba* Mill. by exploring different technology technologies of extraction, isolation, and purification methods, which have determined the different properties and structural features of the jujuba polysaccharides. As the main foundation for the quality evaluation of jujuba polysaccharides, the total sugar contents are commonly determined by phenol sulfuric acid, anthrone-sulfuric acid methods. Homogeneity is the premise of jujuba polysaccharide structure characterization, which is usually represented by using nuclear magnetic resonance (NMR) spectroscopy, high performance liquid chromatography (HPLC), high performance gel permeation chromatography (HPGPC), high-performance size-exclusion chromatography (HPSEC), fourier transform infrared spectroscopy (FT-IR), ultraviolet (UV)-visible spectroscopy, gas chromatography (GC), gas chromatography-mass spectrometry (GC-MS), liquid chromatography-tandem mass spectrometry (LC-ESI-MS), GPC-multi-angle laser light scattering (MALLS), high-performance anion-exchange pulsed amperometric detection chromatography (HPAEC-PAD), X-ray diffraction (XRD), atomic force microscopy (AFM), scanning electron microscopy (SEM), capillary zone electrophoresis (CZE), methylation analysis, periodate oxidation-Smith degradation, and partial acid-hydrolysis, etc. The biological activity of jujuba polysaccharides can be greatly influenced by various factors such as different Mw, composition of monosaccharides, type of linkage, and modification. Herein, the reported jujuba polysaccharide in the past few years are listed and integrated information on their source, Mw, monosaccharide composition, Structural characteristics and bioactivities are comprehensively introduced in Table 2.

**TABLE 2:** Monosaccharide compositions, molecular weights, and structural characteristics of jujuba polysaccharide.

No	Name	Polysaccharides source	Mw (kDa)	Monosaccharide composition	Structural characterization	Analysis technique	Biological activities	Ref
1	HJP1	Ziziphus jujuba Mill (Ziziphus Jujuba cv. Muzao)	6.762	Man: Rha: Gal: GalA: Glu: Ara = 1.3:27.6:6.7:3.7:13:47.6	type I rhamnogalacturonan (containing arabinogalactan/arabinan side chains) domains and typical pectic polysaccharides, with homogalacturonan (methyl and acetyl esterified)	GC-MS, NMR	Antitumor activity	Wang et al. (2015)
2	HJP3	Ziziphus jujuba Mill (Ziziphus Jujuba cv. Muzao)	2.936	Man: Rha: Gal: GalA: Glu: Ara = 0.6:16:16.7:6.5:21:39.2	type I rhamnogalacturonan (containing arabinogalactan/arabinan side chains) domains and typical pectic polysaccharides, with homogalacturonan (methyl and acetyl esterified)	GC-MS, NMR	Antitumor activity	Wang et al. (2015)
3	BJP-2	Z. jujuba cv. Huizao	6.42	GalA: Ara: Gal: Rha: Xyl: GlcA: Glc: Fuc: Man = 39.78: 31.93: 16.86:6.43: 1.86: 1.28:1.02:0.61:0.23	→5)-α-L-Araf (1→4)-β-D-Gal (1→, T-α-L-Araf (1→4)-β-D-Gal (1→, and →4)-α-L-6MeGalAp (1→	DAWN HELEOS-II laser photometer, HPAEC-PAD, GC-MS, NMR	antitumor activity	Zhang et al. (2022)
4	HP1	Zizyphus jujuba cv. Huizao	68.7	Rha: Ara: Man: Glu: Gal = 1.00: 2.43: 3.01: 7.28: 7.11	—	GC, FT-IR	Immunomodulatory	Zou et al. (2018)
5	HP2	Zizyphus jujuba cv. Huizao	111	Rha: Ara: Man: Glu: Gal = 1.00: 3.28: 1.89: 0.48: 2.28	—	GC, FT-IR	Immunomodulatory	Zou et al. (2018)
6	WSPs	Ziziphus jujuba Mill.		Rha: Ara: Gal: Glu: Xyl: GalA = 1.0: 3.6: 1.0: 0.5: 0.2:39.2	—	GC, GC-MS	immunobiological activities	Zhao et al. (2007)
7	JPC	Ziziphus Jujube		Man: Rib: GluA: GalA: Glu: Xyl: Gal: Ara = 5.3: 3.1: 3.6: 11.4: 13.4: 14.5: 23.4: 25.1	—	HPLC, FT-IR	Immunomodulating and antioxidant effects	Chi et al. (2015)
8	ZJMP-2	Zizyphus jujuba Mill.	57.8	Glu: Gal: Ara: Rha: GalA = 0.41:0.08:0.11:0.05:0.33	→2)-*α*-L-Rha*p*-(1 → 4)-*α*-D-Gal*p*A-(1 → 4)-*α*-D-Gal*p*A-6OMe-(1 → 4)-*α*-D-Gal*p*A-(1 → 3, 4)-*α*-D-Glc*p*-(1 →, with branching at →5)-*α*-L-Ara*f*-(1 →, →4)-*β*-D-Gal*p*-(1 →, and →4)-*α*-D-Glc*p*-(1→ at position *O*-3 of →3, 4)-*α*-D-Glc*p*-(1 →	FT-IR, HPGPC-LLS, GC-MS, SEM, AFM, NMR	immunoregulatory activity	Feng et al. (2025)
9	PZMP1	Ziziphus Jujuba cv. Muzao	16.97	Ara: Gal: Glc: Man: Xyl = 17.36:3.29:2.68:1.05:1.00	Backbone composed of 1,3,5-linked Araf, 1,3-linked Araf, and 1,5-linked Araf	HPGPC, GC, FT-IR, XRD, SEM, AFM	Antioxidant activities	Ji et al. (2020a)
10	PZMP2	Ziziphus Jujuba cv. Muzao	62.73	Rha: Ara: Xyl: Gal: GalA = 1.18: 5.23: 0.22: 2.68: 2.20	type I rhamnogalacturonan I (RG-I) with branches at O-4 consisting of oligosaccharide units	HPGPC, GC, FT-IR, XRD, SEM, AFM	Antioxidant activities	Ji et al. (2020a)
11	PZMP3	Ziziphus Jujuba cv. Muzao	58.21	Rha: Ara: Gal: GalA = 1.74: 2.00: 1.00: 18.69	1,4-GalpA residues with some side chains substituted at O-2 position	HPGPC, GC, FT-IR, XRD, SEM, AFM	Antioxidant activities	Ji et al. (2020a)
12	DPZMP3	Z. jujuba cv. Muzao	34.3	Rha: Ara: Gal: GalA = 1.00:1.49:1.60:7.68	homogalacturonan pectic polysaccharide with a (1 → 4)-Galp branch at C-6 and a small amount of Araf and Rhap residues.	HPGPC, FT-IR, and NMR	antioxidant activity	Liu et al. (2024)
13	SAZMP3	Zizyphus jujuba cv. Muzao	9.37	Rha: Ara: Xyl: Man: Glu: Gala: GalA = 10.51: 6.70: 0.50: 0.26: 0.50: 6.75: 74.69	Backbone composed of 1,4-α-D-GalAp with side chains of 1,3-β-D-Galp, 1,3,5-linked Araf, 1,2,4-α-L-Rhap and terminals of 1-linked Araf, 1-linked Rhap, 1 - l i n k e d G a lp	HPGPC, GC, FT-IR, NMR, AFM, SEM	antioxidant activities	Lin et al. (2019)
14	ZSS	Ziziphus jujuba Mill var. spinosa (Bunge) Hu ex H. F. Chou	2.34	GlA: Man: Rha: Glu: Gal: Xyl	rhamnosyl residues were linked with various types (1-linked, 2-linked, 4-linked or 2, 4-linked)	PMP-HPLC, FT-IR, NMR, SEM	antioxidant activity and immunoregulation	Lin et al. (2018)
15	ZP1	Ziziphus jujuba Mill	87.4	Ara: Gal: Glc: Man: Rha: GalA = 10.85:32.05:22.04:1.00:2.73:31.30	—	—	antioxidant activities	Lin et al. (2018)
16	ZP2	Ziziphus jujuba Mill	76.5	Ara: Gal: Glc: Man: Rha: GalA = 4.18:13.04:52.17:1.00:3.66:25.95	—	—	antioxidant activities	Lin et al. (2018)
17	ZP3	Ziziphus jujuba Mill	71.3	Ara: Gal: Glc: Man: Rha: GalA = 12.19:8.36:9.49:16.72:14.27:38.29	(1 → 4)-*α*-D-GalA*p*, (1 → 2,4)- *α*-L-Rha*p*, with a (1 → 4,6)- α-*D*-Gal*p*	UV, FT-IR, HPGPC, GC-MS, NMR	antioxidant activities	Lin et al. (2018)
18	SAZMP	Zizyphus jujuba cv. Muza	9.73	Rha: Ara: Xyl: Man: Glu: Gal: GalA = 10.51: 6.70: 0.50: 0.26: 0.50: 6.75: 74.69	1,4-α-GalAp with side chains of 1,3-β-D-Galp, 1,3,5-linked Araf, 1,2,4-α-L-Rhap and terminals of 1-linked Araf, 1-linked Rhap, 1-linked Galp	HPGPC, GC, FT-IR, NMR	antioxidant activities	Lin et al. (2019)
19	SAZMP4	Ziziphus jujuba cv. Muzao	28.94	Rha: Ara: Xyl: Man: GalA = 1:0.9:0.05:0.07:28.9	1,4-linked GalA (93.48%) with side chains of 1,2,4-linked Rha and 1,3,5-linked Ara and terminals of 1-linked Rha and 1-linked Ara	HPGPC, GC, FI-IR, GC-MS, NMR, SEM, and AFM	antioxidant activities	Lin et al. (2020)
20	BJP-0	Z. jujuba cv. Hamidazao	1.5	Ara: Gal: Glu: Man: Xyl: Fru = 76.71: 11.29: 5.35: 0.78: 4.56: 1.31	—	UV, HPAEC-PAD, FT-IR, NMR	antioxidant activity	Yuan et al. (2022)
21	BJP-1	Z. jujuba cv. Hamidazao	1.36	Ara: Gal: Glu: Man: Xyl: Fru: GalA = 44.46:39.27:7.33:1.92:1.38:2.04:3.59	—	UV, HPAEC-PAD, FT-IR, NMR	antioxidant activity	Yuan et al. (2022)
22	BJP-2	Z. jujuba cv. Hamidazao	13.3	Rha: Ara: Gal: Glu: Xyl: Fru: GalA = 0.43:13.37:61.57:0.71:0.54:0.58:22.80	—	UV, HPAEC-PAD, FT-IR, NMR	antioxidant activity	Yuan et al. (2022)
23	BJP-3	Z. jujuba cv. Hamidazao	11.9	Rha: Ara: Gal: Glu: Man: Xyl: GalA = 10.38:18.88:9.93:1.68:0.08:1.57:57.48	→4)-α-L-GalpA (1→, →5)-α-L-Araf (1→ residues with two terminals of T-α-L-Araf (1→ and T-β-D-Galp (1→.	UV, HPAEC-PAD, FT-IR, NMR	antioxidant activity	Yuan et al. (2022)
24	BJP-4	Z. jujuba cv. Hamidazao	12.3	Ara: Gal: Glu: Man: Fru: GalA = 34.74:15.38:3.02:3.85:9.58:33.42	—	UV, HPAEC-PAD, FT-IR, NMR	antioxidant activity	Yuan et al. (2022)
25	HJP-3	Z. jujuba cv. Hamidazao	6.986	arabinose (24.2%), galactose (11.0%) and rhamnose (9.8%)	→4)-α-D-GalpA (1→ and →2,4)-α-L-Rhap (1→ residues with some branches consisting of →5)-α-L-Araf (1→ residues and terminals of T-α-L-Araf (1→ and T-β-D-Galp residues.	HPAEC-PAD, FT-IR, NMR	antioxidant activities	Yang et al. (2021)
26	ZJP-04M	Ziziphus jujuba Mill	60.26	Rha: Ara: Gal: GalA: Xyl: Glc = 1.00: 4.19: 2.44: 2.50: 0.17: 0.15	homogalacturonan skeleton and rhamnogalacturonan-I domain and the branch chain was formed by arabinan and arabinogalactan	GC-MS, SEM, AFM, HPGPC, NMR	Antioxidant and antiaging activities	Liu et al. (2025)
27		Zizyphus jujube cv. Muzao	89.90	Ara: Gal: Glu, Rha: Man = 4.52: 2.64: 1.04: 0.49: 0.41	—	HPGPC, FT-IR	antioxidant activity	Yan et al. (2018)
28	GZMP-1	Ziziphus Jujuba cv. Muzao	111.2	Rha: Ara: Glu: Gal: GalA = 0.59:72.80:0.26:7.48:6.24	—	HPLC, GC, FT-IR	antioxidant activities	Ji et al. (2018a)
29	GZMP-2	Ziziphus Jujuba cv. Muzao	95.1	Rha: Ara: Gal: GalA = 1.57:29.85:17.05:17.75	—	HPLC, GC, FT-IR	antioxidant activities	Ji et al. (2018a)
30	GZMP-3	Ziziphus Jujuba cv. Muzao	84.2	Rha: Ara: Gal: GalA = 1.68:33.70:5.58:27.41	—	HPLC, GC, FT-IR	antioxidant activities	Ji et al. (2018a)
31	GZMP-4	Ziziphus Jujuba cv. Muzao	571.4	Rha: Ara: GalA = 0.59:2.05:40.81	—	HPLC, GC, FT-IR	antioxidant activities	Ji et al. (2018a)
32	LZJP3	Ziziphus jujuba Mill.	9.766	Gal: alduronic acid = 2.05:6.84	—	FT-IR	antioxidant activities	Wang et al. (2018)
33	LZJP4	Ziziphus jujuba Mill.	5.412	Gal: Glu: alduronic acid = 16.12:3.08:8.16	—	FT-IR	antioxidant activities	Wang et al. (2018)
34	ZMP	Ziziphus jujuba Mill		Rha: Ara: Xyl: Man: Glu: Gal: GalA = 1.46:2.47:2.27:1.12:1.00:1.57:5.40	—	UV, FTIR, and SEM	antioxidant activity	Ji et al. (2018b)
35	—	Ziziphus jujuba Mill. var. inermis (Bunge) Rehd	—	Glu: Xyl: Man: Fru = 23:31.3:12.9:21.6	—	GC-MS, FT-IR	antioxidant activity	Wang (2011)
36	PWJS	Ziziphus jujuba Mill. var. spinosa (Bunge) Hu ex H. F. Chou	—	Man: Rha: GluA: GalA: Glu: Xyl: Gal: Ara = 2.03: 3.74: 1.05: 17.64: 38.59: 3.36 10.44: 23.16	—	HPLC, FT-IR	antioxidant activity	Yue et al. (2014)
37	JCP-1	Ziziphus jujuba Mill.	15	Glu: Fru: Ara: Gal: Rha = 1.4:0.4:2.1:4.2:0.9	—	—	antioxidant activities	Rostami and Gharibzahedi (2016)
38	JCP-2	Ziziphus jujuba Mill.	9.1	Rha: Ara: Gal: Glu = 1.1:1.8:4.1:1.2	—	—	antioxidant activities	Rostami and Gharibzahedi (2016)
39	HJP1	Zizyphus Jujuba cv. Muzao	6.762	Man: Rha: Gal: GalA: Glu: Ara = 4.3:16.4:1.28:7.90:21.8:48.4	—	PMP-HPLC, FT-IR	antioxidative and immunological activities	Zhang et al. (2017)
40	HJP2	Zizyphus Jujuba cv. Muzao	6.13	Man: Rha: Gal: GalA: Glu: Ara = 2.44:4.06:1.42:3.41:55.4:33.3	—	PMP-HPLC, FT-IR	antioxidative and immunological activities	Zhang et al. (2017)
41	HJP3	Zizyphus Jujuba cv. Muzao	2.936	Man: Rha: Gal: GalA: Glu: Ara = 0.69:22.5:6.14:1.68:29.0:40.0	—	PMP-HPLC, FT-IR	antioxidative and immunological activities	Zhang et al. (2017)
42	ZSP1b	Zizyphus jujuba cv. Jinsixiaozao	9.3	Glu		HPGPC, GC, HPSEC	Antioxidant activities	Li et al. (2011)
43	ZSP2	Zizyphus jujuba cv. Jinsixiaozao	8.6	Rha: Ara: Glu: Gal = 1:2.5:1.3:4.1		HPGPC GC, HPSEC	Antioxidant activities	Li et al. (2011)
44	ZSP3c	Zizyphus jujuba cv. Jinsixiaozao	16.0	Rha: Ara: Gal = 1:2:8		HPGPC GC, HPSEC	Antioxidant activities	Li et al. (2011)
45	ZSP4b	Zizyphus jujuba cv. Jinsixiaozao	14.0	Rha: Ara: Man: Gal = 13.8:4:3:8		HPGPC GC, HPSEC	Antioxidant activities	Li et al. (2011)
46	CZPH	Zizyphus Jujuba cv. Junzao	—	Ara: Rha: Glu: Gal: Man = 5.46: 4.89: 3.65: 2.54: 1	—	FT-IR	Antioxidant activity	Li et al. (2014)
47	CZPU	Zizyphus Jujuba cv. Junzao	—	Ara: Rha: Glu: Gal: Man = 5.46: 4.96: 5.17: 2.63: 1	—	FT-IR	Antioxidant activity	Li et al. (2014)
48	ZJRP	Ziziphus jujuba cv. Muzao	—	Rha: Ara: Glu: Gala: GluA: galA = 3.3:3.0:2.6:1.9:1.0:25.1	—	FT-IR, GC	Antioxidant activities	He et al. (2019)
49	ZSP	Zizyphus jujube cv. Shaanbeitanzao		Man: Rib: Rha: GluA, GalA: Glu: Xyl: Gal: Ara = 2.8: 1.8: 6.6: 2.6: 10.9: 5.3: 3.4: 16.5: 50.2		PMP-HPLC	Antioxidant activity	Wang et al. (2012)
50	ZJPs-II	Ziziphus jujuba (Z. jujube cv. Ruoqiangzao)	115	Ara: Rha: Glu: Xyl: Gal = 26.31: 8.62: 18.35: 15.72: 5.52	—	HPGPC, FT-IR, NMR	Antioxidant and antitumor activities	Wu et al. (2021)
51	HJP	Zizyphus jujube cv. Huanghetanzao	—	Man: Rha: GalA: Glu: Gal: Ara = 2.62: 14.3:8.40: 5.29: 32.9: 36.4	—	PMP-HPLC	Hepatoprotective effects	Liu et al. (2015a)
52	ZSP	Zizyphus jujube cv. Shaanbeitanzao	—	Xyl: Ara: Glu: Rib: Rha: Gal: Man: GluA: GalA = 2.8: 52.0: 5.5: 1.3: 7.2: 14.4: 2.0: 2.5: 8.8	—	capillary zone electrophoresis (CZE)	Anti-hyperglycemic or antihyperlipidemic	Zhao et al. (2014)
53	WJPs	Ziziphus jujuba Mill. var. spinosa (Bunge) Hu ex H. F. Chou	—	Ara glu (38.59%): Ara (23.16%): GalA(17.64%): Gal (10.44%)		HPLC	Anti- inflammatory bowel diseases	Yue et al. (2015)
54	ZJP	Ziziphus jujuba Mill (Zizyphus jujuba cv. Junzao)	153.3	GalA	1,4-linked D-Gal*p*A	GPC-MALLS, LC-ESI-MS, GC–MS, NMR, FT-IR	Anti-inflammatory effects	Zhan et al. (2018)
55	ZY-2	Ziziphus jujuba Mill. var. Spinosa (Bunge) Hu ex H. F. Chou	7.76	Man: Rha: GluA: GalA: Glu: Gal: Xyl: Ara = 7.22:8.54:3.89:2.32:4.89:20.56: 39.67:6.3	α-type and β-type glycosidic bonds with obvious triple helix structural traits	HPLC, FT-IR, NMR	Anti-inflammatory effects	Liu et al. (2023)
56	ZY-3	Ziziphus jujuba Mill. var. Spinosa (Bunge) Hu ex H. F. Chou	10.71	Man: Rha: GluA: GalA: Glu: Gal: Xyl: Ara = 6.11:7.32:2.22:4.15:7.18:35.28:54.17:10	α-type and β-type glycosidic bonds with obvious triple helix structural traits	HPLC, FT-IR, NMR	Anti-inflammatory effects	Liu et al. (2023)
57	ZY-4	Ziziphus jujuba Mill. var. Spinosa (Bunge) Hu ex H. F. Chou	8.31	Man: Rha: GluA: GalA: Glu: Gal: Xyl: Ara = 7.28:7.47:3.89:5.15:10.16:18.89:44.66:5	α-type and β-type glycosidic bonds with obvious triple helix structural traits	HPLC, FT-IR, NMR	Anti-inflammatory effects	Liu et al. (2023)
58	JP-H	Ziziphus jujuba Mill.	107.09	Man: GluA: Rha: GalA: Glu: Gal: Xyl: Ara = 2.19:1.53:1.89:50.60:7.37:16.89:1.08:20.53	→5)-α-L-Araf-(1→, →3)-α-L-Araf-(1→,→3)-β-Galp (l→, →4)-β-L-GalpA (1→	HPLC, FT-IR, NMR	Prebiotic activity	Zou et al. (2022)
59	JP-U	Ziziphus jujuba Mill.	92.342	Man: GluA: Rha: GalA: Glu: Gal: Xyl: Ara = 2.11:2.22:1.51:50.64:5.78:14.52:2.36:20.85	→5)-α-L-Araf-(1→, →3)-α-L-Araf-(1→,→3)-β-Galp (l→, →4)-β-L-GalpA (1→, →4)-α-D-Glcp-(1→	HPLC, FT-IR, NMR	Prebiotic activity	Zou et al. (2022)
60	JP-D	Ziziphus jujuba Mill.	72.212	Man: GluA: Rha: GalA: Glu: Gal: Xyl: Ara = 1.31:2.27:0.92:68.72:2.71:12.48:0.65:10.93	→5)-α-L-Araf-(1→, →3)-α-L-Araf-(1→,→3)-β-Galp (l→, →4)-β-L-GalpA (1→	HPLC, FT-IR, NMR	Prebiotic activity	Zou et al. (2022)
61	JP-UD	Ziziphus jujuba Mill.	72.99	Man: GluA: Rha: GalA: Glu: Gal: Xyl: Ara = 1.17:2.64:1.02:60.46:2.02:13.26:0.59:18.84	→5)-α-L-Araf-(1→, →3)-α-L-Araf-(1→,→3)-β-Galp (l→, →4)-β-L-GalpA (1→, →4)-α-GalpA-(1→	HPLC, FT-IR, NMR	Prebiotic activity	Zou et al. (2022)
62	AJP1	Jinsixiaozao	75.465	Rha: Ara: Man: Xyl: Gal: Glu = 0.1:0.31:0.36:0.16:0.01:0.20	—	GC-MS, FT-IR, SEM	Regulating gut microbiota	Wang et al. (2023)
63	AJP2	Jinsixiaozao	26.8678	Rha: Ara: Man: Rib: Xyl: Gal: Glu = 0.78:1.11:1.36:0.13:0.25:0.51:0.18	—	GC-MS, FT-IR, SEM	Regulating gut microbiota	Wang et al. (2023)
64	JP	Zizyphus jujuba Mill. Goutouzao	242	GalA: Ara: Gal: Rha: Glu: xyl = 4.2:1.0:0.6:0.5:0.2:0.2		HPAEC-PAD, FT-IR, NMR	Anti- bacteria	Xu et al. (2022)
65	PZMP3-1	Ziziphus Jujuba cv. Muzao	241	Rha: Ara: Gal: GalA = 2.56:7.70:3.73:6.73	1,2,4 and 1,4-linked GalpA, 1,4-linked Galp, 1,3 and 1,5-linked Araf, and 1-linked Rhap	XRD, FI-IR, AFM, SEM	—	Ji et al. (2022)
66	PZMP2-2	Ziziphus Jujuba Mill	62.73	Rha: Ara: Xyl: Gal: GalA = 1.18: 5.23: 0.22: 2.68: 2.20	→ 4)-GalpA-(1 → 2,4)-Rhap-(1→, with branches at O-4 consisting of Araf and Galp residues	FT-IR, SEM, AFM, GC-MS, NMR	—	Ji et al. (2019c)
67	PZMP3-2	Zizyphus jujuba Mill.	58.21	Rha: Ara: Gal: GalA = 1.74:2.00: 1.00:18.69	→4)-Gal*p*A-(1→residues backbone, with few branches at the *O*-2 position of some Araf and Rhap residues	HPGPC, FT-IR, GC-MS, NMR	—	Ji et al. (2020b)
68	ZP2a	Zizyphus jujuba cv. Junzao	12.06	Rha: Ara: Glu = 1.3:1.7:0.3:1	1,4-d-GalpA residues interspersed with 1,2-l-Rhap and 1,2,4-l-Rhap residues. The branches were composed of 1,5-l-Araf, 1,3,5-l-Araf, 1,3-l-Araf,1,6-d-Galp, 1,4,6-d-Galp and 1,4-d-Glcp. The branches were attached to the backbone at the O-4 position of Rhap residues	GC–MS, FT-IR, NMR	—	Li et al. (2013)
69	ZJSPs-1	Z. jujube cv. Ruoqiangzao	342	arabinose, glucose, xylose, galactose and rhamnose = 21.63:9.81:13.52:15.28:8.73	pyranose-form sugars as well as both α- and β-configurations	FT-IR, NMR, XRD, AFM, SEM		Wu et al. (2019)
70	ZP2a	Zizyphus jujuba cv. Junzao	12.06	Rha: Ara: Glu: Gal = 1.3:1.7:0.3:1	1,4-D-GalpA residues interspersed with 1,2-l-Rhap and 1,2,4-L-Rhap residues, Branch chain: 1,5-L-Araf, 1,3,5-L-Araf, 1,3-L-Araf,1,6-D-Galp, 1,4,6-D-Galp and 1,4-D-Glcp	GC-MS, FT-IR, NMR	—	Li et al. (2013)
71	PZMP4	Ziziphus jujuba Mill (Ziziphus Jujuba cv. Muzao)	27.90	Rha: Ara: Man: Glu: Gal: GalA = 2.32:2.21:0.22:0.88:2.08:8.83	(1→4)-linked Gal*p*A with three branches bonded to *O*-3 of (1→3)-linked Ara*f* (1→2)-linked Rha*p*, and terminated with Gal*p*A	HPGPC, GC, FT-IR, NMR, SEM, AFM	—	Ji et al. (2021a)

Rhamnose (Rha), Arabinose (Ara), Fucose (Fuc), Mannose (Man), Glucose (Glu), Galactose (Gal), Glucuronic acid (GluA), Galacturonic acid (GalA), Ribose (Rib), Fructan (Fru).

### 3.1 Relative molecular mass

Mw of jujuba polysaccharide is a key parameter that needs to be considered in understanding their biological activities and functions, thus determination of the Mw of polysaccharides can help determine the optimal range of biological activities and provide guidance for further research and application. The Mw of jujuba polysaccharide is currently determined using HPGPC, HPSEC, HPLC. Differentiation of average Mw of jujuba polysaccharides vary across studies due to variations in plant source, extraction techniques, purification methods, and analytical approaches. For instance, the JP-H, JP-U, JP-UD and JP-D reported come from *Zizyphus jujube* cv. Hui-zao using different extraction methods, with high and low Mw in the order of JP-H (107.09 kDa) (HWE) > JP-U (92.342 kDa) (UPE) > JP-UD (72.99 kDa) (UPADESE) > JP-D (72.212 kDa) (DESE) (Zou et al., 2022). In addition, Mw fraction of *Z. jujuba* cv. *Junzao* polysaccharide obtained by HWE (ZJP) (153.3 kDa), was higher than that of *Z. jujuba* cv. *Junzao* polysaccharide obtained by HWE (ZP2a) (12.06 kDa) (Li et al., 2013; Zhan et al., 2018). As shown in Table 2, different experimental conditions observed that the Mw of jujuba polysaccharides ranged from 1 to 100 kDa.

### 3.2 Monosaccharide composition

The polysaccharides found in *Ziziphus jujuba* Mill plants exhibit significant variations in monosaccharide composition. These differences can be attributed to the variations in extraction, separation, purification methods, and detection techniques, leading to inconsistencies in the composition and proportion of monosaccharides. However, it can be seen from Table 2 that the monosaccharide composition of various jujuba polysaccharides was different, but most of them were composed of mannose (Man), rhamnose (Rha), glucose (Glu), galactose (Gal), xylose (Xyl), arabinose (Ara), glucuronic acid (GluA), and galacturonic acid (GalA), and the proportion of each component was different. It is worth noting that fructose (Fuc) main in *Z. jujuba* cv. Huizao polysaccharides were found, which is mainly composed of GalA, Ara, Gal, Rha, Xyl, GlcA, Glc, Fuc, Man with a molar ratio of 39.78: 31.93: 16.86:6.43: 1.86: 1.28:1.02:0.61:0.23 (Zhang et al., 2022). In addition, ribose (Rib) main in Ziziphus. jujuba cv. Jinsixiaozao and Shaanbeitanzao were found. It is worth noting that some jujuba polysaccharides are homogeneous. Moreover, significant differences in the monosaccharide composition and molar ratio of jujuba polysaccharides obtained from same extraction method and different purification processes have been observed. For instance, a crude jujuba polysaccharides CZSP were extracted from Zizyphus jujuba cv. Jinsixiaozao (Li et al., 2011). Four kinds of jujuba polysaccharides ZSP1b, ZSP2, ZSP3c and ZSP4b were obtained by DEAE-SepharoseCL-6B and SepharoseCL-6B column chromatography methods. The results showed that the composition of four water-soluble polysaccharide components ZSP1b, ZSP2, ZSP3c, and ZSP4b was different.

### 3.3 Chemical structures

**FIGURE 3:**
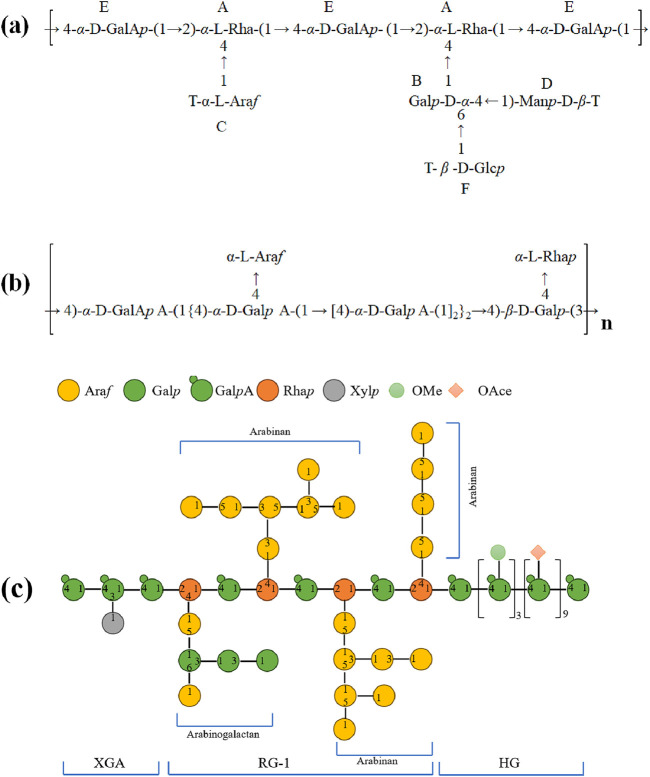
Summary of the typical structural features of diverse polysaccharides from *Ziziphus jujuba* Mill. **(a)** ZP3; **(b)** JP; **(c)** ZJP-04M.

The biological activity of polysaccharides is intimately related to their structure, which includes the branch chain, configuration, and mechanism of connection of the glycosidic bond. Nevertheless, according to the existing literatures, the chemical structure of jujuba polysaccharides is rarely mentioned in literature reports. Researchers have attempted to categorize and examine the chemical structure of the current jujuba polysaccharides in an effort to close this knowledge gap and gather more useful data. By using this categorization analysis method, we intend to uncover some possible traits and attributes of jujuba polysaccharides and offer more detailed recommendations for subsequent research. Liu et al. (2020b) extracted a water-soluble ZP3 from *Ziziphus jujuba* Mill. fruit. The structures of the compounds were determined by UV, IR-RT, ^1^H NMR and ^13^C NMR spectra. It was found that the purified ZP3 were mainly composed a main chain of (1→4)-*α*-D-GalA*p*, (1→2,4)-*α*-L-Rha*p*, with a (1→4,6)-*α*-D-Gal*p* branches. A homogeneous acidic polysaccharide (PZMP4) was isolated from *Ziziphus jujuba* Mill. by Ji et al. (2021b) in their study. AFM and SEM analysis results showed that PZMP4 was a netted structure of molecular aggregates. Using a combination of HPGPC, GC, FT-IR, GC-MS, NMR, a comprehensive analysis of PZMP4 was conducted. The results revealed that the main chain of PZMP4 mainly composed of (1→4)-linked Gal*p*A, with three branches boned to *O*-3 of (1→3)-linked Ara*f*, (1→2)-linked Rha*p*, and terminated with Gal*p*A. In addition, Zhan et al. (2018) extracted a pectin polysaccharide with anti-inflammatory activity and found that they had 1,4-linked D-Gal*p*A. Wang et al. (2015) deciphered the chemical structures of two pectin like HJP1 and HJP3, revealing that both of them have a type I rhamnolactouronic acid (containing arabinogalactan/arabinan side chain) domain and a typical pectin polysaccharide. Pectin is classified into Xylogalacturonan (XGA), Homogalacturonan (HG), and Rhamnogalacturonan-I (RG-I) regions. Similarly, Zou et al. (2022) isolated with four different extraction techniques, and purified four polysaccharides (JP-H, JP-U, JP-D and JP-UD) from *Ziziphus jujuba* Mill. Through modern instrumental analysis methods, including HPLC, FT-IR and NMR, each refined JP-H, JP-U, JP-D and JP-UD was analyzed, and its primary structure was inferred. NMR results showed that four polysaccharides contained similar glycosidic linkage of →5)-*α*-L-Ara*f*-(1→, →3)-α-L-Ara*f*-(1→, →3)-*β*-Gal*p*-(1→ and →4)-*β*-L-Gal*p*A-(l→, while the specific glycosidic linkage of →4)-α-D-Glc*p*-(l→ appeared in JP-U and JP-UD, the esterified units of galacturonic acid and the CONH_2_ group appeared in JP-D and JP-UD, as well the Terminal β-D-Gal*p* and →4)-α-Gal*p*A-(1→ appeared in JP-UD. The structural characterization of jujuba polysaccharide were summarized as presented in Table. 2, and the potential structures of polysaccharide were depicted in Figure 3.

### 3.4 Molecular morphology

At present, SEM, AFM and XRD have been widely used to determine the molecular morphological properties of jujuba polysaccharides. Among them, SEM is the most commonly used method for determining the morphology of polysaccharide molecules, which can perform continuous slicing and imaging of the sample, and then use computer software packages for segmentation and three-dimensional (3D) reconstruction to achieve visualization of the three-dimensional structure. Wang et al. (2023) used SEM to observe the microstructure of AJP1 and AJP2, and found that AJP2 has a smooth surface and dense sheet-like interior, while the surface of AJP1 is relatively rough, loose, and porous. The results showed that the loose pores between AJP1 polysaccharides were more likely to bind with water, increasing the surface area of polysaccharides and improving their solubility. Another study used SEM and AFM to image PZMP1, PZMP2 and PZMP3 obtained by HWE extraction methods. At ×10,000 magnification, SEM found that PZMP1, PZMP2, and PZMP3 have different surface morphologies. Among them, PZMP1 is in an aggregated state, appearing as thin flakes with a smooth surface, while PZMP2 has a spherical network structure. PZMP3 has a rough surface with flocculent fibers and different branch chains. They inferred that the different microstructural features are mainly due to the different monosaccharide compositions of the three PZMPs, especially the different content of uronic acid and glycosidic bonds links. In addition, at a magnification of 5,000 times, AFM observation revealed a clear spherical mass in PZPP1, indicating molecular aggregation. PZMP2 constituted of random linear chains with a small number of spherical aggregates, forming a spherical structure as a whole, while PZMP3 dispersed in extremely dilute solutions could be exhibit monodisperse, spherical, and irregular particles. They inferred that the different microstructural characteristics can be attributed to differences in hydrogen bonding and galacturonic acid content (Ji et al., 2020a).

## 4 Biological activities of jujuba polysaccharides

**FIGURE 4:**
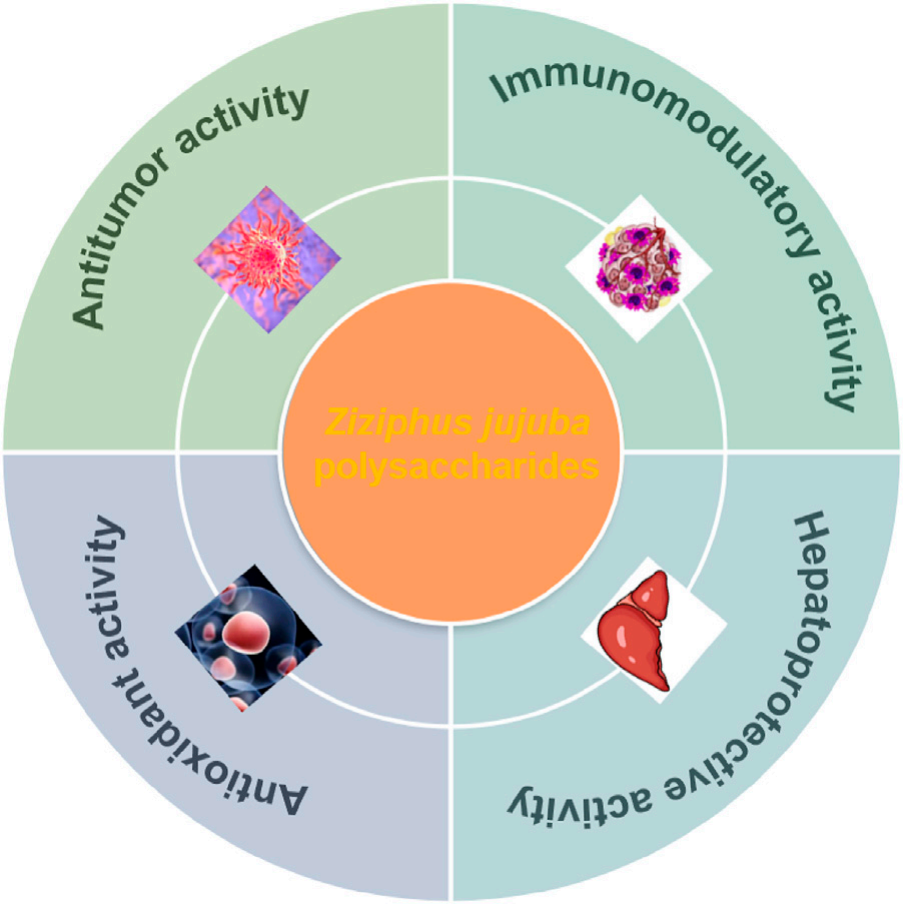
The health benefits of jujuba polysaccharide.

The bioactivities of jujuba polysaccharide from their unique structural characteristics, which promote interactions with biological systems and mechanisms of action that contribute to the potential health benefits. Jujuba polysaccharide exhibit anti-cancer, hepatoprotective effects, anti-oxidant, anti-inflammatory, anti-bacterial, anti-hyperglycemic, regulating gut microbiota and immunomodulatory effects which have attracted significant interest from researchers worldwide. The diverse bioactivity of jujuba polysaccharides highlights their potential as natural agents for the management and prevention of a range of illnesses as well as their role in the creation of novel medical goods and treatments in the future. Figure 4 shows the combined health benefits.

### 4.1 Antitumor activity

Cancer is a major global public health issue and the third leading cause of death globally (Li et al., 2024). The latest data reported that approximately 2,001,140 new cancer cases and 611,720 cancer deaths the United States in 2024 (Siegel et al., 2024). The medical community still seeks the causes of cancer and effective treatments, as current chemical drugs therapy methods often cause harm and approved drugs have significant side effects, emphasizing the urgent need for safer anti-tumor therapies. Medicinal plant polysaccharides, as natural active ingredients, are used to inhibit tumor cell growth through various molecular mechanisms, including: 1) preventing tumor invasion and metastasis by lowering tumor cell adhesion or trophic factor levels; 2) stimulating T and NK cells to play an immune role in preventing cancer growth; 3) decreasing tumor cell viability or displaying cytotoxic effects; 4) preventing cancer cell division and proliferation by modifying the expression levels of genes and proteins involved in the cell cycle; 5) inducing mitochondrial dysfunction and antioxidant system imbalance, and increasing cell apoptosis mediated by cysteine protease (Xue et al., 2024). So far, compared to the researches provided for other polysaccharides, there are few findings presented on the anti-tumor activity of jujuba polysaccharides. Wang et al. (2015) prepared the water-soluble polysaccharides (HJP1 and HJP3) by UAE and found that HJP1 and HJP3 exhibited a significant inhibitory effect on human HepG2 cells *in vitro*. Additionally, the significantly stronger cytotoxicity of HJP3 against HepG2 cells compared to HJP1 suggests that both HJP1 and HJP3 have potential as natural anti-tumor candidates. Wu et al. (2021) extracted Ziziphus jujuba polysaccharides (ZJPs) using UAE, and then purified the crude ZJPs to obtain homogeneous polysaccharides fractions (ZJPs-II). The results observed that ZJPs-II could effectively inhibit the proliferation of SW620 cells, arresting them in the G2/M phase, reducing the cell colony-formation, and inducing apoptosis and arrested. Zhang et al. (2022) found that BJP-2 could induce apoptosis in a dose-dependent manner, leading to the inhibition of cell proliferation. In addition, cell scratching and Trans well assays demonstrated that BJP-2 could effectively inhibit the invasion and metastasis of tumor cells. Moreover, BJP-2 could upregulate the expression of Bax, Cleaved Caspase-3/Caspase-3 and Cleaved Caspase-9/Caspase-9 and downregulation of Bcl-2, suggesting that the anti-tumor activity of BJP-2 is mediated through mitochondrial dependent pathways. Most importantly, HJP3 with lower molecular weight (2.94 kDa) exhibited greater anti-tumor activity than HJP1 (6.76 kDa). The structure-function relationship indicated that type I rhamnogalacturonan (containing arabinogalactan/arabinan side chains) domains and typical pectic polysaccharides, with homogalacturonan (methyl and acetyl esteried), thereby exerting remarkable anti-tumor activity. Additionally, the special structure with the backbone of the main chain of →5)-α-L-Araf (1→4)-β-D-Gal (1→, T-α-L-Araf (1→4)-β-D-Gal (1→, and →4)-α-L-6MeGalAp (1→, is also of great importance for promoting the anti-tumor effect.

### 4.2 Immunomodulatory activity

Natural polysaccharides are well known to have better immunomodulatory properties. Han et al. (2020) found that JP could improve the immune response and restore intestinal barrier function in immunosuppressed mice (induced by cyclophosphamide). In comparison with the model group, JP (150, 300, and 600 mg/kg BW per day) significantly increased the lymphocyte proliferation in the spleen and decrease the proportion of CD3^+^ and CD4^+^ and the ratio of CD4+/CD8+ in cyclophosphamide-induced mice in a dose-dependent manner after 28 continuous days of treatment. In addition, JP treatment in immunosuppressed mice also increased the levels of IL-2, IL-4, IL-10, IFN-*γ*, and TNF-α in serum and the intestine, and the improvement effects were proportional to the dose of JP. JP at a dose of 600 mg/kg BW demonstrated better immune activation. *In vitro*, JP significantly enhanced the proliferation of T lymphoma cells, and they triggered significant changes in immune cells at concentrations as low as 150 *μ*g/mL. Zou et al. (2018) found that both medium and high concentrations of HP1 and HP2 (100 and 200 mg/kg BW per day) could significantly increase spleen and thymus indices, promote serum hemolysin formation, enhance the phagocytic activity of macrophages and inhibit footpad edema of mice after seven consecutive days of treatment. HP2 at a dose of 200 mg/kg BW demonstrated better immune activation. It is worth noting that JPC can also promote NK cell activities, T cell proliferation, CD4^+^/CD8^+^ ratio and CD4^+^ counts in CFS rats, thereby improving the body and helping restore immune function (Chi et al., 2015). Extensive researches have displayed the anti-inflammatory and immunomodulatory effects of polysaccharides were closely related to their structures, especially the Mw, monosaccharide composition. HP-2 with a relatively higher molecular weight (111 kDa) than HP-1 (68.7 kDa) and higher ratio of galacturonic acid (35.9%) than HP-1 (7.32%) significantly exert immunomodulatory activity.

Conversely, ZJMP-2, a relatively ratio of galacturonic acid (0.33%), with a very small Mw of 57.8 kDa and the backbone comprised →2)-α-L-Rhap-(1 → 4)-α-D-GalpA-(1 → 4)-α-D-GalpA-6OMe-(1 → 4)-α-D-GalpA-(1 → 3, 4)-α-D-Glcp-(1 →, with branching at →5)-α-L-Araf-(1 →, →4)-β-D-Galp-(1 →, and →4)-α-D-Glcp-(1→ at position O-3 of →3, 4)-α-DGlcp-(1 →, significantly promoted the expression levels of TLR4, NF-*κ*B, and TRAF6 proteins, increasing RAW264.7 cell activity, index of splenic lymphocytes, and the production of cytokines and NO, thereby activating macrophages and elevating lymphocyte proliferation. So far, the relationship of jujuba polysaccharides structure with the immunomodulatory function is still unclear. Further research on the structure-activity relationship of jujuba polysaccharides will be beneficial for the development and application of Z. jujuba polysaccharides as a immunomodulatory drug.

### 4.3 Antioxidant activity

Reactive oxygen species (ROS) have been implicated in the development of multiple diseases, including hypertension, diabetes, aging and cancer (Li et al., 2021). The antioxidant properties of Jujuba polysaccharides are closely linked to their ability to neutralize ROS and inhibit lipid peroxidation. This action helps protect cells and tissues from oxidative damage, maintains cellular redox balance, and may enhance overall cellular health while mitigating conditions related to oxidative stress. Research on the antioxidant activity of Jujuba polysaccharides has shown promising results and potential therapeutic benefits.

*In vitro* antioxidant activity is typically assessed through straightforward methods, including the measurement of free radical and reduce Fe^2+^ (Yu-Hao et al., 2021). He et al. (2019) optimized the alkali extraction process to obtain a water-soluble ZJRP from the *Ziziphus jujuba* cv. Muzao residue. Their study shown that ZJRP have a strong antioxidant effect at a lower concentration (1 mg/mL) and can effectively scavenge DPPH free radicals, with scavenging rate of 70.67%. However, their antioxidant activities were not surprising when compared to the corresponding concentration of ascorbic acid. They speculated that the antioxidant activity of ZJRP may be related to high levels of galacturonic acid and extraction methods. Guo et al. (2024) found that the JPS obtained by HWE, UAE, EAE and UAEE had the ability to scavenge hydroxyl radicals, ABTS radical, reducing power and DPPH free radical and exhibited a reducing capacity in a dose-dependent manner. The results of DPPH, ABTS and hydroxyl free radicals scavenging experiments showed that JPS-UAEE had the strongest scavenging ability on three kinds of free radicals, and the scavenging effects were 77.98%, 91.82% and 66.85%, respectively. Additionally, absorbance represents reducibility, and the larger the reduction capacity, the higher the anti-oxidant capacity. The reducing capacities and ABTS radical scavenging ability of JPS obtained from UAEE higher than that of EAE, UAE and HWE. However, the extraction of JPS using UAEE method significantly reduced the IC50 value compared to other samples, which may be due to the fact that UAEE in these frequency modes induced more severe degradation of JPS during the extraction process, changed the Mw, thus enhancing hydrogen ion donor ability and DPPH scavenging efficiency. Research indicates that polysaccharides with lower Mw exhibit higher antioxidant activity. Liu et al. (2024) investigated the antioxidant activity of three jujuba polysaccharide fractions (DPZMP3, DZMP, and ZMP) extracts (i.e., purified polysaccharides, degraded polysaccharides, and crude polysaccharides) obtained from *Ziziphus Jujuba* cv. *Muzao*. The oxidation ability of different polysaccharide fractions was found to be concentration-dependent. When the concentration of DPZMP3, DZMP, and ZMP used was 2.0 mg/mL, the scavenging rates of DPPH radical reached 92.89%, 89.64%, and 81.55%, respectively. In a specific concentration range, the DPPH radical scavenging ability of ZP1, ZP2 and ZP3 (extracted using subcritical water and purified using DEAE-52 anion-exchange chromatography) were 74.8%, 64.9%, and 88.3%, respectively. Further structure-activity relationship indicated DPZMP3 had a smaller Mw of 34.3 kDa than ZP1 (874 kDa), ZP2 (765 kDa), and ZP3 (713 kDa), and was mainly composed of Rha, Ara, Gal, and GalA, which may be related to the fact that after the treatment, the molecular chain of polysaccharide is broken, and more active groups are exposed to the solution, making them easier to create contact between active sites and free radicals, thus exerting the anti-oxidant activities.

The most commonly utilized *in vivo* models for investigating antioxidant activity are normal mice and *C. elegans*. The activities of antioxidant enzymes and the content of ROS are used to measure the ability of polysaccharides to reduce oxidative damage in *C. elegans* models. Han et al. (2024) isolated and purified a polysaccharide (CPJE) with a molecular weight of 98 kDa from *Ziziphus jujuba* Mill cv. *Jinsixiaozao*. CPJE can significantly reduce the levels of ROS, and increased the activities of SOD, CAT, and the content of GSH. The research indicates that CPJE can regulate the transcription factors DAF-16 and SKN-1 to modulate oxidative stress in *Caenorhabditis elegans*, and can also exert an antiaging role by mediating DAF-16 and SKN-1 pathways and inhibiting IIS pathways in nematode model. In addition, the homogeneous polysaccharide ZJP-04M, isolated and purified from *Ziziphus jujuba* fruit, exhibits significant anti-aging and antioxidant effects. It also exerts an anti-aging role by alleviating oxidative stress in nematodes and modulating the downstream target of the conserved insulin/IGF-1 signaling pathway (IIS), FOXO homolog DAF-16. Further structure-activity relationship indicated ZJP-04M had Mw of 60.26 kDa, and was mainly composed of Rha, Ara, Gal, and GalA, with small amounts of Xyl and Glu, which may be related to a fairly long linear HG skeleton was alternately connected with RG-I to form the trunk chain, and multibranched arabinan and AG domains coexist in the C4 site of Rhap on the trunk chain, thus exerting the anti-oxidant and antiaging activities (Liu et al., 2025).

Further investigation reveals that the anti-oxidant effect of *Z. Jujuba* polysaccharides is mainly associated with the Mw, monosaccharide content, glycosidic bond type, and uronic acid content. Based to the published literature (Table 2), *Z. Jujuba* polysaccharides with molecular weights ranging from 1.36 kDa to 571.4 kDa exhibited remarkable anti-oxidant action. Notably, the antioxidant activity of polysaccharides is proportional to their galacturonic acid, glucose, galactose, arabinose, fucose, mannose, xylose content, plays an important role in the anti-oxidant impact of *Z. Jujuba* polysaccharides. In general, the antioxidant activity of polysaccharides is connected to their uronic acid content due to more functional groups including -COOH and -OH in these monosaccharides, which can offer more hydrogen ions to neutralize unpaired electrons in free radicals. For instance, ZSP3c and ZSP4b comprising higher galacturonic acid content had the stronger free radical scavenging activities than ZSP1b including no uronic acid. The antioxidant impact of PZMP3 higher than PZMP2 and PZMP1 by neutralizing unpaired electrons in free radical activity due to their functional groups, including higher galacturonic acid groups. Additionally, the structure-function relationship indicated BJP-1 with the highest content of arabinose (44.46%) and lowest molecular weight (1.36 kDa) exhibited great anti-oxidant activity than other compounds by scavenging DPPH and ABTS radicals. The polysaccharides with a low-molecular weight tend to possess strong antioxidant activity due to more reducing hydroxyl groups at their terminals.

Other monosaccharides, in addition to uronic acid, also play an important role in the antioxidant activity of polysaccharides. The content of glucose affected the *in vitro* antioxidant activity of jujuba polysaccharide fractions against hydroxyl radical and ferric-reducing.

Furthermore, various factors influence the antioxidant activity of polysaccharides. Polysaccharide biological activities are thus determined by interactions between various structural variables, most notably monosaccharide content and molecular weight.

The structure-activity relationship revealed that the antioxidant effect of polysaccharides was closely related to its structure. structural characteristics demonstrated Galacturonic acid occupied the terminal region of the branched chains and attached to the main chain, connecting by α-(1→4) bonds at *O*-4 and *O*-6 position and accompanying by arabinose, xylose, rhamnose, galactose and glucose residues, thereby exerting remarkable anti-oxidant activity. So far, despite the abundance of literature on the anti-oxidant effect of jujuba polysaccharides, research on the structure-activity relationship of *Z. jujuba* polysaccharides is still obscure, and it requires immediate attention to elaborate comprehensively, which provides a strong basis to further investigate the functional food or anti-oxidant medicine of *Z. jujuba* in the coming years.

### 4.4 Hepatoprotective activity

Carbon tetrachloride (CCl_4_) administration catalyzes the formation of reactive trichloromethyl radicals (CCl_3_) via cytochrome P450, which subsequently convert to trichloromethyl peroxide radicals (CCl_3_OO), precursors of lipid peroxidation. This lipid peroxidation leads to liver damage, resulting in the release of alanine aminotransferase (ALT) and aspartate aminotransferase (AST) (McCay et al., 1984; Yang et al., 2010). Consequently, serum levels of ALT and AST are important indicators of liver injury. The polysaccharides from jujuba have been reported to have protective effect on liver injury (Gao et al., 2013; Liu et al., 2015a). For example, Sun et al. (2022) investigated the potential health benefits of WJP-F80, particularly *in vivo* assessment of protective effects against CCl_4_-induced liver injury in mice. The results indicate that the prophylactic treatment with WJP-F80 before CCl_4_ administration showed the ability to decrease the levels of both ALT and AST in a dose-dependent manner by comparing with the CCl_4_-model group. At a dose of 100 mg/kgBW, the ALT level decreased sharply, while the AST levels had no significant statistical difference. At a dose of 200 body weight of WJP-F80, the activities of ALT and AST were close to those of the normal control group, and that 400 mg/kg·WJP-F80 significantly reduced the CCl_4_-elevated levels of ALT and AST. Histopathological analysis further demonstrated that WJP-F80 treatment improved liver histological integrity in a dose-dependent manner. Mice receiving low-dose WJP-F80 exhibited only mild inflammatory cell infiltration, whereas high-dose treatment preserved normal liver architecture, effectively preventing cell necrosis and inflammatory infiltration.

At present, comprehensive researches on the hepatoprotective effects of jujuba polysaccharides are lacking. Existing theories suggest that their liver-protective functions may involve scavenging free radicals and enhancing antioxidant enzyme activity. Therefore, further detailed investigations are needed to elucidate the hepatoprotective activities of jujuba polysaccharides.

### 4.5 Other bioactivities

Except for the aforementioned pharmacological effects, jujuba polysaccharides also exhibit various physiological activities, including antihyperglycemic, anti-hyperlipidemic, prebiotic activity, anti-bacteria, and regulating gut microbiota. An earlier study revealed that jujuba polysaccharides can significantly reduce the serum levels of glucose, insulin, TC, TG, LDL-C, and VLDLC, improve the HDL-C level, homeostasis model assessment for insulin resistance (HOMA-IR), b-cell function (HOMA-b), and decrease the atherogenic index (AI) of the mice exposed to high-fructose water, and address hyperglycemic or hyperlipidemic (Zhao et al., 2014). Furthermore, Xu et al. (2022) investigated the mechanism of jujuba polysaccharides in preventing and treating on oral biofilm pathogens by human saliva collection. Their findings indicate that jujuba polysaccharides can significantly inhibit the activity of oral biofilm pathogens *Streptococcus mutans*, MRSA, *Porphyromonas gingivalis* and prevent and treat oral infectious diseases.

Jujuba polysaccharides demonstrate significant potential for functional food applications due to their diverse bioactive properties. In the formulation of functional foods that incorporate jujuba polysaccharides, it is crucial to address several key factors, including formulation stability, bioavailability, and potential interactions with other ingredients. For example, due to its dietary fiber content and prebiotic properties, Jujuba polysaccharides has the potential to promote digestive health. Furthermore, their prebiotic effects can promote the growth of beneficial gut bacteria, thereby enhancing gut microbiota composition. Given these attributes, jujuba polysaccharides represent a promising ingredient for functional foods and dietary supplements aimed at promoting human health and preventing diseases (Wang et al., 2023; Zou et al., 2022).

Furthermore, the prebiotic actions of Jujuba polysaccharides may indirectly contribute to immune support by encouraging the growth of beneficial gut bacteria involved in immune system regulation and function. Jujuba polysaccharides have the potential to have prebiotic effects, which encourage the development and reproduction of beneficial gut bacteria. As a prebiotic, Jujuba polysaccharides act as a substrate for some beneficial microbes in the gut, promoting their growth and potentially improving gut microbiota composition. Jujuba polysaccharides may help to improve the health and diversity of the gut microbiota by supporting these beneficial gut bacteria. Jujuba polysaccharides prebiotic actions suggest that they could be used in functional foods, nutritional supplements, or probiotic formulae to promote gut health.

## 5 Conclusion and perspectives

As a homolog of medicinal and edible food, *Z. jujuba* fruit has been widely exploited for functional food, dietary supplement and therapeutic medicines owing to its excellent nutritional and medicinal benefits, as well as its range of therapeutic effects and health-promoting properties. This review summarized the extraction, separation, and purification, structure identification, and biological functions research on polysaccharides from *Ziziphus Jujuba* Mill. It highlights the crucial potential of jujuba polysaccharides as precious natural compounds and advocates for their further exploration and application in both industry and therapeutics. Previous researches have addressed that the content of jujuba polysaccharides varies with different extraction techniques and purification techniques. jujuba polysaccharides have been shown to have a variety of potential health benefits, including antioxidant activity, immunomodulatory function, antitumor activity, hypoglycemic and hypolipidemic effects, modulation of gut microbiota, prebiotic activity, and antiaging activities, among others, making them beneficial for the development of novel therapeutics.

Despite these potential characteristics, present research identifies several areas requiring further exploration: (1) The yield, purity, stability, reproducibility and massive production of jujuba polysaccharides is difficult to guarantee due to the limitations of current extraction methods, and the interaction of polysaccharides with other components of the food matrix is not fully understood during the extraction process. Therefore, the researchers may confront with challenges in improving these processes to acquire high-purity jujuba polysaccharides for their investigations; (2) Numerous investigations prioritize extraction yield optimization over detailed characterization of the resulting jujuba polysaccharides, especially its structure and functionality. The specific chemical structure and composition of jujuba polysaccharides remain difficult to determine. Jujuba polysaccharides is a complex plant polysaccharide, which extraction process is affected by varieties of differences and extraction methods, resulting in the significant differences in polysaccharide structure. All of these variables contribute to the difficulty of the development of jujuba polysaccharides-based drugs. (3) Though multiple pharmacological activities of jujuba polysaccharides have been confirmed by diverse experiments *in vivo* and *in vitro* in animals, the mechanisms underlying the biological functions have not been completely clarified. Compared with other polysaccharides, investigation on the structural characterization and structure-activity relationship of jujuba polysaccharides is still in the early stages. (4) The research on jujuba polysaccharides demonstrating pharmacological activities primarily focuses on cell models and animal experiments. However, the safety evaluation, toxicity, efficacy, pharmacokinetics, and clinical trials of jujuba polysaccharides have not been thoroughly elucidated, which are essential prerequisites for the development of functional foods, nutritional supplements, and pharmaceuticals.

Future research on jujuba polysaccharides should focus on the following objectives: including the development of the innovative extraction and purification techniques; the optimization of production processes for commercialization; the continued efforts to elucidate structural features for the better understanding of bioactivities and potential mechanisms of jujuba polysaccharides; the conduction of the *in vitro* and *in vivo* experiments in animals and clinical studies with regards to antioxidant activity, immunomodulatory function, antitumor activity, hypoglycemic and hypolipidemic effects, modulation of gut microbiota, prebiotic activity of jujuba polysaccharides.

In conclusion, t this review systematically summarizes the research progress on the preparation, structural characteristics, and biological activities of jujuba polysaccharides. A more detailed understanding of the structure, activity and structure-activity relationship of jujuba polysaccharides will offer valuable insights and references to facilitate their development and application in functional foods, nutritional supplements, and pharmaceuticals.
